# Book Review: Interpersonal Interactions and Language Learning: Face-To-Face vs. Computer-Mediated Communication

**DOI:** 10.3389/fpsyg.2021.710234

**Published:** 2021-06-21

**Authors:** Hui Zhao

**Affiliations:** ^1^School of Chinese Language and Literature, Shandong Normal University, Jinan, China; ^2^School of Literature and Journalism, Weifang University, Weifang, China

**Keywords:** F2F (face-to-face), communication, second language acquisition, interpersonal interaction, language learning

The use of synchronous and diachronous technology has been exponentially surging lately and has been dramatically and abruptly augmented by the unprecedented Covid-19 pandemic that has coerced EFL/ESL educators and students to struggle over online classes. With the recognition of computer-mediated communication (CMC), online discussions have also gained tremendous momentum in the teaching and learning process. Given that learners are the key stakeholders in this process, their individual characteristics such as personality, motivation, anxiety, confidence, as well as language proficiency among so many other factors need to be carefully considered. Therefore, *Interpersonal Interactions and Language Learning: Face-to-Face vs. Computer-Mediated Communication*, co-authored by Shin Yi Chew and Lee Luan Ng, is a timely contribution that focuses on the synchronous text-based CMC where language learners with diverse individual differences and language proficiency levels communicate with each other by typing their messages which are read by other users on computer screens synchronously.

Collectively, this monograph consists of seven chapters. Chapter 1 foregrounds the crucial role of communication in second language acquisition by scrutinizing the historical and theoretical developments of learning approaches and justifying the growing popularity of communicative language teaching. The chapter also succinctly elaborates on some of the germane theories in communication, elucidates how technology has brought about some changes in the Malaysian context, and accentuates the potential of collaborative discussion as a communicative learning task in the L2 classroom because “discussions can serve three important roles for participants: to ask for information, clarify matters and share information” (p. 8).

Chapter 2 makes a demarcation between face-to-face (F2F) and online communication by describing such communication models as (a) the simplest model which involves the sender and receiver of the message; (b) Shannon's (1948) information theory model that demonstrates the way communication takes place between a sender and receiver in telecommunication; (c) the intermediary model that constitutes a gatekeeper who has the ability to “choose, decide and even change what messages to be received by the audience” (p. 18); (d) the interactive or cybernetic model adds the cybernetic notion of feedback, so the sender of the message can modify or correct it; (e) the transactional model which expands communication to other interactive media such as emails, radio, and letters; and (f) Foulger's (2004). Ecological Model of Communication which pertains to the dynamic and multifaceted nature of communication between the interlocutors.

Chapter 3 comprehensively makes a distinction between CMC and F2F communication. The authors stipulate that CMC and F2F interactions differ in terms of communication skills, language, collaboration, turn-taking, and nonverbal cues. Furthermore, the chapter overviews the pertinent early theories and models of the effective use of communication such as social presence theory, the lack of social context cues hypothesis, and media richness theory as well as some current theories and models, including common ground theory, media synchronicity theory, social information processing (SIP) theory, hyperpersonal model of CMC, efficiency framework, and ICT succession framework. The chapter finishes with some viable pedagogical implications of CMC in language classrooms.

Focusing on learning individual differences (IDs), namely learners' personality and language proficiency, is the objective of Chapter 4. The chapter also brings to the fore the impact of these two IDs on the learners' participation styles, lexical complexity, and interactive competence of learners with diverse personalities across different levels of proficiency when they are engaged in discussions in conventional F2F and synchronous online text-based discussion. Furthermore, the authors opine that since learners are complex and dynamic, we need to explore the impact of other IDs such as motivation, confidence, and language anxiety. The chapter problematizes that we need to understand the potential disruptions and noises in different discussion environments, so we know how to make ourselves ready to tackle the contingent problems.

The penultimate chapter encapsulates the pedagogical implications and some recommendations for language instructors and discussion facilitators who are involved in group discussion activities. It is highly suggested that teachers need to be strategic to deal with idiosyncratic individuals with various emotional, cognitive, social, and academic characteristics. For instance, it is recommended that language teachers “gain a better understanding of the learners in terms of their strengths, weaknesses, aims, interests, expectations, motivations” (p. 86), through establishing some benchmarks for students' behavior in group discussions, and “carry out online text-based discussions to promote balanced participation in a mixed classroom” (p. 87).

The final chapter offers a short and sweet overview of the current advancements in the employability of technology in communication and language learning. More specifically, the chapter highlights the advantages of multimodality in communication, mobile-mediated communication (MMC), computer-assisted language learning, online video conferencing, and online gaming. The chapter rightly cautions us that “despite the benefits technology has to offer, we need to use it carefully due to the potential distractions it carries” (p. 96), and it should be judiciously and strategically utilized for different students to realize and optimize its full potential.

The present monograph could have been more insightful if the authors had elaborated on massive open online courses (MOOC) which have been one of the prevailing modes of CMC. Moreover, in Chapter 2, the authors highlighted the role of cognitive learning theories and lost sight of including (Vygotsky's, 1986) sociocultural theory and its related approaches, highlighting that interaction, negotiation, collaboration, and culture play an indispensable role in communication. However, regardless of these pitfalls, I recommend this didactic and timely contribution to EFL/ESL students, teachers, teacher educators, and syllabus designers who are interested in IDs and CMC.

## Author Contributions

The author confirms being the sole contributor of this work and has approved it for publication.

## Conflict of Interest

The author declares that the research was conducted in the absence of any commercial or financial relationships that could be construed as a potential conflict of interest.
